# A novel allele of *FILAMENTOUS FLOWER *reveals new insights on the link between inflorescence and floral meristem organization and flower morphogenesis

**DOI:** 10.1186/1471-2229-10-131

**Published:** 2010-06-28

**Authors:** Nitsan Lugassi, Naomi Nakayama, Rachel Bochnik, Moriyah Zik

**Affiliations:** 1 Department of Life Sciences, Ben Gurion University, Beer - Sheva 84105, Israel; 2 Department of Molecular, Cellular and Developmental Biology, Yale University, New Haven, CT, 06520-8104, USA; 3 Institute of Plant Sciences, University of Bern, 3013 Bern, Switzerland

## Abstract

**Background:**

The *Arabidopsis FILAMENTOUS FLOWER (FIL) *gene encodes a YABBY (YAB) family putative transcription factor that has been implicated in specifying abaxial cell identities and thus regulating organ polarity of lateral organs. In contrast to double mutants of *fil *and other *YAB *genes, *fil *single mutants display mainly floral and inflorescence morphological defects that do not reflect merely a loss of abaxial identity. Recently, *FIL *and other *YABs *have been shown to regulate meristem organization in a non-cell-autonomous manner. In a screen for new mutations affecting floral organ morphology and development, we have identified a novel allele of FIL, *fil-9 *and characterized its floral and meristem phenotypes.

**Results:**

The *fil-9 *mutation results in highly variable disruptions in floral organ numbers and size, partial homeotic transformations, and in defective inflorescence organization. Examination of meristems indicates that both *fil-9 *inflorescence and floral meristems are enlarged as a result of an increase in cell number, and deformed. Furthermore, primordia emergence from these meristems is disrupted such that several primordia arise simultaneously instead of sequentially. Many of the organs produced by the inflorescence meristems are filamentous, yet they are not considered by the plant as flowers. The severity of both floral organs and meristem phenotypes is increased acropetally and in higher growth temperature.

**Conclusions:**

Detailed analysis following the development of *fil-9 *inflorescence and flowers throughout flower development enabled the drawing of a causal link between multiple traits of *fil-9 *phenotypes. The study reinforces the suggested role of *FIL *in meristem organization. The loss of spatial and temporal organization of *fil-9 *inflorescence and floral meristems presumably leads to disrupted cell allocation to developing floral organs and to a blurring of organ whorl boundaries. This disruption is reflected in morphological and organ identity aberrations of *fil-9 *floral organs and in the production of filamentous organs that are not perceived as flowers. Here, we show the role of *FIL *in reproductive meristem development and emphasize the potential of using *fil *mutants to study mersitem organization and the related effects on flower morphogenesis.

## Background

The shoot apical meristem (SAM) consists of a relatively small population of pluripotent cells, proliferation and allocation of which gives rise to the entire above ground plant body. The SAM is organized into three zones, namely a central zone (CZ) at the SAM summit in which cells divide slowly to replenish the SAM population, a slowly-dividing organizing center (OC) which lies below the CZ, and a peripheral zone (PZ) in which cells divide more rapidly and become allocated to presumptive organ primordia [1]. SAM homeostasis is dependent on the appropriate balance of cell proliferation and differentiation among these three zones.

These meristematic domains are maintained in part by a negative feedback loop between *WUSCHEL *(*WUS) *and *CLAVATA3 *(*CLV3*) (for recent reviews, see [2-4]). *WUS *encodes a homeodomain-containing transcription factor that is expressed in the OC and acts to promote the expression of the CLV3 ligand in the CZ [5]. CLV3 is thought to bind to the CLV1/CLV2 receptor complex, which in turn limits the domain of expression of *WUS *[6]. The indeterminate state of cells within the meristem is also dependent on members of the *KNOX *gene family that are expressed in the SAM but excluded from incipient organ primordia [7].

Cells at the periphery of the CZ begin to differentiate concomitant with the downregulation of *KNOX *genes and the expression of *AINTEGUMENTA *(*ANT*), regulating cell proliferation in the emerging organ primordia [8,9]. Organ outgrowth is accompanied by the establishment of a boundary zone separating the primordium from the adjacent meristematic tissues [2]. Lateral organ polarity is determined by the converse activities of abaxially-expressed *KANADI *and members of the *YABBY *(*YAB*) gene family and of the adaxially-expressed *PHABULOSA *and *PHAVOLUTA *genes [10]. SAM activity is also regulated by signals emanating from the organ primordia. Several studies have demonstrated that abaxialization of organs by ectopic expression of abaxial genes or by repression of adaxial genes causes arrest or loss of the SAM [10-13]. Additionally, in *Petunia*, the *HAIRY MERISTEM *gene that is expressed in organ primordia is required for SAM maintenance [14].

Upon transition from the vegetative to the reproductive phase, the SAM becomes an inflorescence meristem (IM). The IM gives rise to lateral meristems in its PZ which acquire a floral fate to become floral meristems (FMs). The FMs differentiate into flowers composed of four whorls of different types of floral organs. In *Arabidopsis*, specific combinations of the four classes of organ identity genes (ABCE) specify the type of organ formed in each flower whorl. The ABCE factors are expressed specifically in the whorls of their function, with spatial specificity of their expression domains being critical for correct floral organ differentiation and for avoiding the appearance of chimeric organs with mixed identities [15].

Proper flower development demands maintenance of the borders between the whorls within the flower and between the organs in each whorl. A and C class factors negatively regulate each other, thereby creating mutually exclusive expression domains divided at the second and third whorl boundary. *SUPERMAN *(*SUP*) regulates the border between the third and forth whorls, preventing expansion of B factors into the forth whorl [16]. UNSUAL FLORAL ORGANS (UFO) activates the B class genes found in the second and third whorls and is thought to set up the inner and outer boundaries of the B domain [17,18]. *CUP SHAPED COTYLEDON1/2 *regulate the separation of organs within whorls (sepals and stamens) [19,20] and are also expressed in the borders of whorl 2, where they inhibit proliferation of cells in these regions [21].

In a mutant screen for plants affected in floral organ development, we have isolated a new allele of *filamentous flower *(*fil*), a member of the *YAB *(*YAB*) gene family. The *YAB *genes, *FILAMENTOUS FLOWER (FIL), YAB2 *and *YAB3*, are all expressed on the abaxial side of developing vegetative lateral organ primordia [12,22-24]. *FIL *is expressed in a dynamic fashion during floral development, with expression initially seen throughout the abaxial side of floral meristems, and later on the abaxial sides of floral organ primordia [22]. *fil yab3 *double mutants exhibit a loss of adaxial-abaxial polarity in the vegetative organs and occasional formation of ectopic SAM structures on leaves [12]. Yet, *fil *single mutants do not show a vegetative phenotype [12,25-27] but rather are strongly affected in flower structure and floral organ number, morphology and to some extent, identity.

*fil *mutant plants also show abnormalities in inflorescence development. As *fil *floral phenotypes can not be explained in terms of loss of abaxial identity, a different role for *FIL *in inflorescence and flower development is likely. Earlier studies, in addressing the range of *fil *phenotypes, had indicated that *FIL *plays multiple roles in inflorescence and flower formation and development [25-28]. Recently, it was suggested that *FIL*, together with *YAB3 *and other genes, regulates the organized growth of the SAM via a non-cell-autonomous mechanism [24,29] . By driving expression of *YAB *family members in the PZ of the SAM, alterations in the expression of CZ markers could be detected, implying that *YAB *genes function in signaling across the meristem [24]. *FIL *itself is redundantly regulated by the myb domain gene, *ASYMMETRIC LEAVES (AS1)*, and the trans-acting siRNA gene, *TAS3*, both of which are expressed in organ primordia [30]. In turn, genes expressed in the meristem also control *FIL *expression [23].

In this study, we have isolated a new *fil *allele, *fil*-9, and dissected the process of reproductive organ development in the mutant. By following flower development from the inflorescence and floral mersitems, we have discovered new features of *fil *phenotypes. We observe a correlation between the meristem phenotypes to different aspects of *fil *floral mutants. As such, we suggest a causal link between the role of *fil *in meristem organization and the multifaceted floral and inflorescence abnormalities.

## Results

### Identification of a novel *fil *allele

To identify new mutations affecting floral organ morphology and development, we screened a population of activation tagged T-DNA lines [31]. This screen yielded a mutant whose phenotype resembled that of *fil *loss-of-function plants [25-27]. When crossed with *fil-8 *[27,32] homozygous plants, all F1 progeny showed the *fil *mutant phenotype, indicating that the newly isolated mutant was allelic to *fil*. Therefore the novel mutant was named *fil-9*. *fil-9 *backcrosses yielded a 3:1 ratio of wild type versus *fil *mutant phenotypes in the F2 generation (92 wild type:29 mutant), indicating that the *fil-9 *mutation segregated as a single locus recessive mutation. In addition, the *fil-9 *line was outcrossed for three generations to eliminate any possible additional mutations.

**Figure 1:**
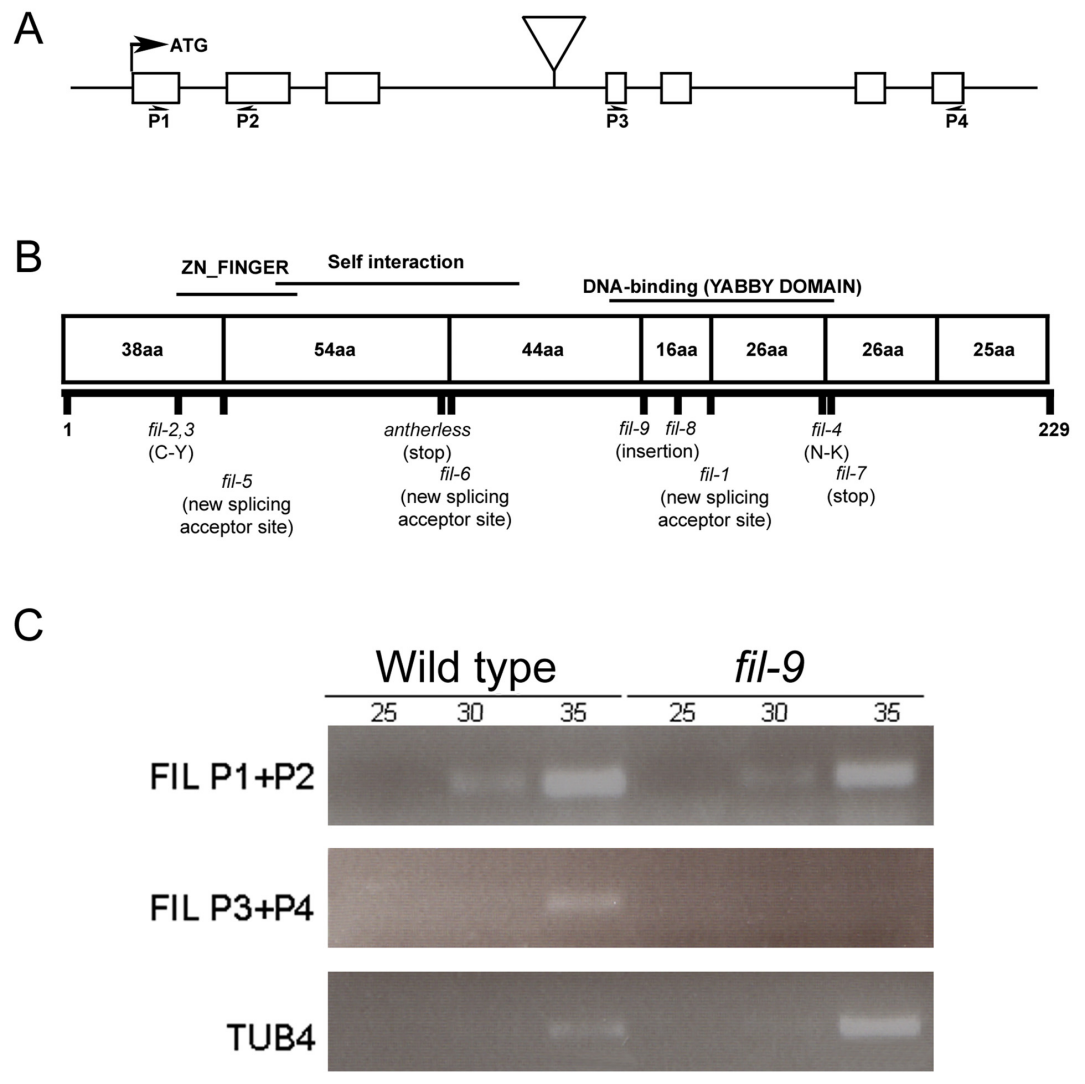
**The T-DNA insertion in the *fil-9 *allele**. (A) Genomic structure of *FIL *showing the T-DNA insertion in the *fil-9 *allele (triangle). Boxes correspond to exons and the black lines to introns. (B) Schematic representation of FIL protein structure. Boxes represent protein domains encoded by different exons. FIL functional domains: Zinc (ZN)-Finger domain (aa 30-57), self-interaction domain (aa 45-107) and DNA-binding domain (YABBY; HMG-related [26,52]) (aa 120-180), according to ExPASy [33], are designated above the protein structure. The locations of the T-DNA insertion or the amino acid changes in different *fil *mutant alleles are indicated below the protein structure. (C) RT-PCR analysis of *FIL *mRNA levels in *fil-9*. Top panel, PCR analysis was performed using *FIL *primers P1 and P2 (location indicated in A) to detect the transcript encoded by the region upstream to the T-DNA insertion. Middle panel, PCR analysis was performed using *FIL *primers P3 and P4 (location indicated in A) to detect that part of the transcript encoded by the region downstream to the T-DNA insertion. Lower panel, the *TUB4 *mRNA level was used as a control. The numbers above the lanes indicate the number of PCR cycles performed.

The T-DNA insert was mapped to the third intron of the *FIL *gene (Figure 1A), using T-DNA- and gene-specific primers (see Methods). RT-PCR analyses using gene-specific primers (Figure 1A) spanning the region upstream to the T-DNA insert or the region downstream to the insertion were performed to verify that the T-DNA insertion indeed disrupts *FIL *gene expression. As demonstrated by RT-PCR, a partial transcript was produced, albeit at a somewhat lower level than in wild type. The full length transcript was not, however, detected (Figure 1C). The *FIL *region upstream to the T-DNA insertion contains the coding sequence of the zinc finger domain of the protein (amino acid residues 22-60; [26] as well as the putative self-interaction domain (amino acid residues 22-60; [33] but lacks the HMG-box DNA binding domain (amino acid residues 146-179; [26]; Figure 1B). Although a partial *FIL *transcript is produced by the *fil-9 *allele, the mutant phenotypes indicate that the T-DNA insertion most likely results in *FIL *loss-of-function and does not cause a dominant negative effect. Finally, it should be noted that *fil-9 *is in the Columbia-7 (Col-7) background [31], while all other characterized *fil *alleles are in the Landsberg *erecta *(L*er*) background [12].

### *fil-9 *affects floral form

**Figure 2:**
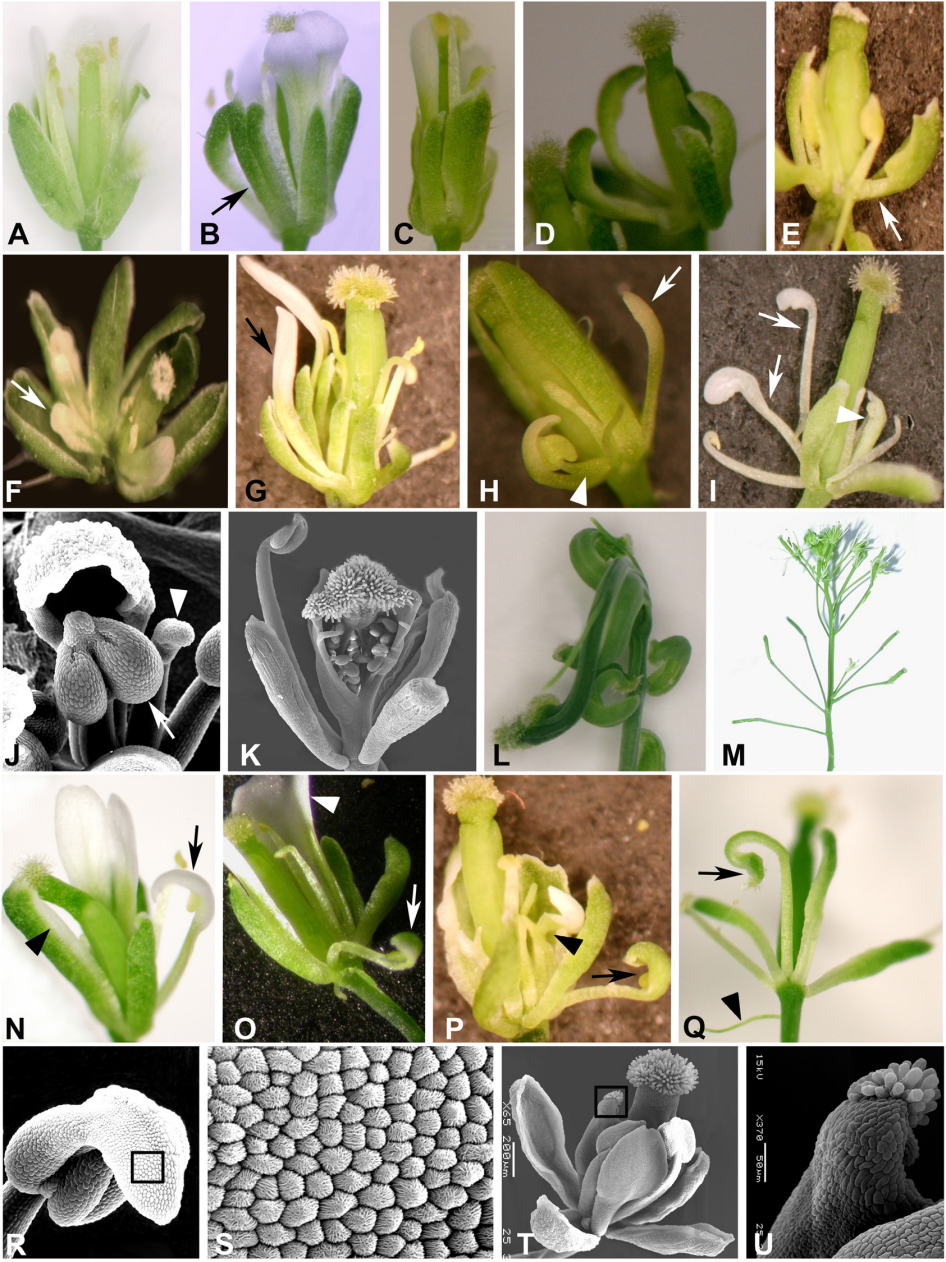
***fil-9 *flowers show a wide range of organ morphological and identity defects**. (A) *Arabidopsis *wild type flower. (B to U) *fil-9 *mutant flowers (B to K and N to U, see text for detailed description), terminating inflorescence (L) and flowers along the inflorescence stem (M). Arrow in B indicates a split sepal, in E, a sepal with a filamentous base, in F, a short petal, and in G a narrow petal. Arrow and arrowhead indicate, respectively: A narrow petal and a stamen fused at the base to a sepaloid petal in H, filaments bearing a petaloid blade and a stamen fused at the base to a sepaloid petal in I, an enlarged, swollen anther and a flattened bent anther in J, a sepal with petaloid margins and a stamenoid petal in N, an enlarged petal and a sepaloid petal in O, a petal with a filamentous base and a sepaloid organ containing a stamen-like locule in P, and a filament toped by stigmatic tissue and a bract subtending a flower in Q. (R) A filament harboring a mosaic organ composed of an anther and a petal. (S) The cells of the petaloid tissue (shown by the box in R) are the conical cells that are characteristic of the petal epidermis. (T) The organ, surrounded by a box and seen in a higher magnification in U, resembles a stamen filament bearing stigmatic tissue at the edge. J, K and R to U are scanning electron micrographs.

Plants homozygous for *fil-9 *generated flowers with a highly complex and variable phenotype (Figure 2), such that each flower presented a different combination of organ types. These phenotypes consisted of alterations in organ number, morphology, and position (summarized in Table 1). It is noteworthy that most aspects of the mutant phenotype were more severe in later-arising flowers (i.e. flower 11 and later) than in early flowers (i.e. flowers 1-10). The number of sepals in the first whorl of many *fil-9 *flowers was either increased (five to seven sepals instead of four, Figure 2B) or decreased. Some of the later-arising flowers contained split sepals that remained fused at the base (Figure 2B, arrow). Other morphological defects included small sepals (Figure 2C), narrow and inwardly curled sepals (Figure 2D), and rarely, sepal-like blades on a narrow-filamentous base (Figure 2E).

**Table 1:** Morphological defects in *fil*-9

Organ	Phenotype	Percentage of flowers (%)		
		**Early flowers (1-10)**^**a**^	**Late flowers (≥ 11)**^**b**^	**Total**^**c**^
		
Sepals	Narrow	9	13	12
	Fused	0	14	9
	Small	9	28	20
	Curled	1	7	5
	Filamentous base	0	3	2
	Increased number (> 4)	23	31	28
	Reduced number (< 4)	10	28	23
Petals	Narrow	13	9	10
	Wide	0	2	1
	Small	6	28	21
	Curled	6	9	8
	Filamentous base	10	9	9
	Filamentous	7	10	9
	Increased number (> 4)	3	0	1
	Reduced number (< 4)	23	86	68
Stamens	Undeveloped anther	14	27	23
	Swollen anther	3	10	8
	Bent anther (hook-like)	6	1	3
	Filamentous	31	55	48
	Attached to sepals	3	1	1
	Increased number (> 6)	3	0	1
	Reduced number (< 6)	53	100	86
Gynoecium	Unfused carpels	1	5	4
	Three carpels	0	2	1
	Distorted style	1	1	1

^a ^Number of flowers scored was 70 (7 plants).

^b ^Number of flowers scored was 162 (7 plants).

^c ^Number of flowers scored was 232 (7 plants).

The phenotype of the second whorl was most apparent, since reduction in petal number was noted from the first flowers. The first few flowers usually developed one or two petals (Figures 2B and 2C), while most late-arising flowers lacked petals (Figures 2D and 2E). Many of the petals were shorter or narrower than were wild type petals, and some had a filamentous base or were completely filamentous (Figures 2F-2I). In contrast to the general reduction in petal size, petals wider than those seen in the wild type also developed, albeit rarely (Figure 2O).

Functional stamens (i.e. pollen-producing) developed almost only in early-arising flowers (i.e. within the first five flowers), with the number of stamens being highly variable in *fil-9 *flowers. Determining the number of stamens was, however, difficult. Many filamentous structures that developed were most likely stamens in origin. However, it was often unclear from which whorl the filaments originated (Figures 2G-2I). Some of the stamens developed an anther presenting normal morphology yet that did not mature to shed pollen. Many of the stamens bore a distorted anther which could range from containing enlarged and swollen locules (Figure 2J, arrow) to assuming a flattened, elongated and curled structure (Figure 2J, arrowhead). Often, only a filament developed, with its edge being sometimes swollen and bent. Occasionally, stamens developed at the base of, or attached to, a petal (Figure 2H) or a sepal (Figure 2I).

Most *fil-9 *flowers developed normal carpels, yet only the first few flowers were fertile and produced seeds. Manual pollination with wild type pollen was successful, indicating that the semi-sterility of the plants resulted from the presence of mostly dysfunctional stamens. Nonetheless, in later-arising flowers, some carpels were not fully fused and the septum was not fully united (Figure 2K), some contained bent carpels, and in some, the style was elongated (Figure 2N). In a few cases, the gyneocium comprised three carpels. Very late flowers (i.e. starting approximately from the 35^th ^flower) were composed only of a gyneocium, which was highly twisted (Figure 2L). This appearance corresponds to a characteristic termination form of *Arabidopsis *plants that fail to develop seeds [34].

*fil-9 *flowers thus showed a general reduction in organ number and size. However, the number of sepals and, occasionally, carpels was increased, while different organ types were occasionally enlarged, in comparison to wild type organs.

### *fil-9 *floral organ identity defects

*fil-9 *flowers displayed an array of partial homeotic transformations in sepals, petals and stamens (Table 2). However, homeotic transformations occurred in less than a quarter of all flowers. Notably, these transformations were never complete, instead resulting in chimeric organs with mixed identity, with our definition of a chimeric organ not being based on the organ shape (e.g. a filamentous base). Instead, we considered an organ to have a chimeric identity only if it displayed tissue-characteristics of different organ types, i.e. sepals with characteristics of petal epidermis, etc. The most common transformed organs were petaloid sepals (usually sepals with white petaloid margins; Figure 2N, arrowhead). Very rarely, sepals with stigmatic tissue at the edge (i.e. carpeloid sepals) were observed (data not shown). Petals or petaloid organs developing locules (stamenoid petals) were also relatively frequent (Figure 2N). Much rarer were sepaloid petals (petaloid organs in the second whorl with a green-sepaloid tip; Figure 2O, arrow) or sepaloid organs containing locules in the second whorl (Figure 2P, arrow). Occasionally, *fil-9 *flowers contained stamens in which the anther elongated into a distal structure composed of conical petal epidermis cells (Figures 2R and 2S), or stamen-like filaments harboring stigmatic tissue (Figures 2T and 2U).

**Table 2:** Organ Identity defects in *fil*-9

Organ	Phenotype	Percentage of flowers (%)		
		**Early flowers (1-10)**^**a**^	**Late flowers (≥ 11)**^**b**^	**Total**^**c**^
		
Sepals	Petaloid	17	25	22
	Carpeloid	0	1	0
Petals	Stamenoid	11	19	16
	Sepaloid	4	1	2
Stamens	Carpeloid	3	0	2
	Petaloid	0	2	1

^a ^Number of flowers scored was 70 (7 plants).

^b ^Number of flowers scored was 162 (7 plants).

^c ^Number of flowers scored was 232 (7 plants).

Overall, the changes in identity of *fil-9 *floral organs were variable and did not implicate simple reduction or ectopic expression of a specific class of organ identity genes. The partial transformation of sepals to petals suggested an expansion of B class function, while the formation of stigmatic papillae on stamen-like organs was indicative of a loss of B class activity. Transformation of petals into a more stamenoid structure and the development of locules on petals implied the expansion of C class function. On the other hand, some of the mosaic organs appeared to be stamens transformed into petals indicative of a loss of C class function. Thus, it appears that these partial homeotic transformations are the result of variable shifts in the domains of organ identity gene activity.

In addition to the marked floral defects, *fil-9 *plants showed other abnormalities reflecting defects in inflorescence development. These include elongated pedicels, the occasional development of a filamentous bract subtending the flower (Figure 2Q), and the clustering of flowers in relatively short segments along the inflorescence, separated by segments harboring short green filaments (Figure 2M).

The variable phenotype of *fil-9 *flowers suggested that the severity of the effect of the mutation was somewhat stochastic. The possibility that growth temperature could have an effect on the severity of mutant phenotype was thus tested. When *fil-9 *plants were grown at a higher temperature (i.e. 28°C versus 22°C), severe organ defects appeared in earlier-arising flowers, as compared to the wild type. In addition, the percentage of flowers containing organs with mixed identity was higher at 28°C (25%), as compared to the percentage obtained at 22°C (19%).

### *fil-9 *morphological defects can be traced to the inflorescence meristem

**Figure 3:**
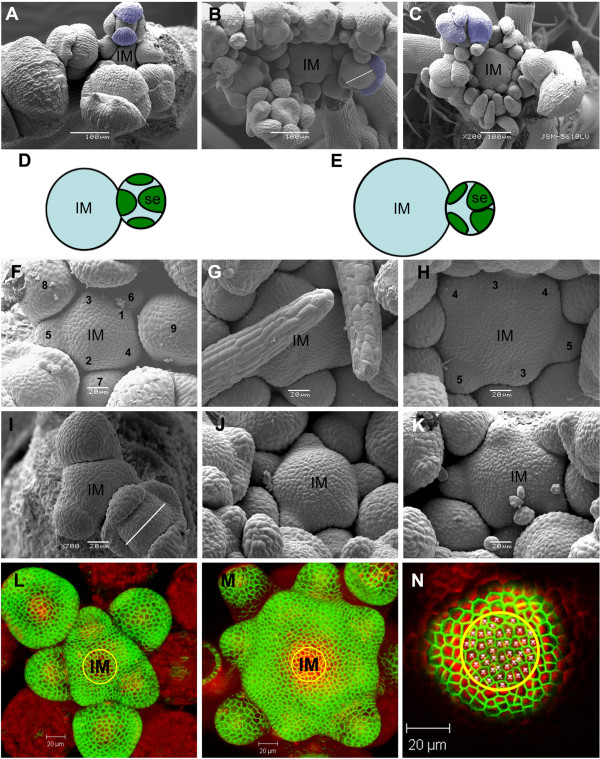
***fil-9 *versus wild type inflorescence meristems and floral primordia**. (A to C) Top view of early wild type (A) and *fil-9 *(B, C) inflorescences. The scale bar represents 100 μm. (D and E) A schematic representation of floral orientation relative to the IM in wild type and *fil-9 *plants, respectively. IM, inflorescence mersitem; Se, sepal. (F to H) Enlarged view of early wild type (F) and *fil-9 *(G, H) inflorescences. Floral primordia are labeled 0, 1, 2, etc., in order of increasing age. The scale bar represents 20 μM. (I to K) Enlarged view of late wild type (I) and *fil-9 *(J, K) inflorescences. The scale bar represents 20 μm. IM, inflorescence mersitem. (L to N) Top view of early wild type (L) and *fil-9 *(M and enlarged view in N) inflorescences, labeled with FM4-64 (red) PIN1-GFP (green). The scale bar represents 20 μm. The yellow circle designates the area used for cell number count. The average number of cells in the defined meristematic area did not differ between *fil-9 *and wild type plants (ANOVA, F_1,38 _= 0.326, P = 0.571).

The overall reduced number and size of *fil-9 *floral organs raised the possibility that these defects were caused by changes in meristem size. Therefore, *fil-9 *inflorescence apices were compared to those of wild type plants by SEM (Figure 3, Table 3). IMs from two developmental stages were analyzed. Early meristems were defined as those containing only two mature open flowers (Figures 3A-3C and 3F-3H), while late meristems were defined as those that had experienced the maturation of twenty flowers (Figures 3I-K).

**Table 3:** The average size of inflorescence meristem

	22°C				28°C			
	Early meristem mean ± SE (μm)		Late meristem mean ± SE (μm)		Early meristem mean ± SE (μm)		Late meristem mean ± SE (μm)	
Col-7	n = 9	52 ± 1.7	n = 7	36 ± 1.8	n = 9	57 ± 1.4	n = 8	41 ± 1.3
*fil*-9	n = 9	77 ± 3.1	n = 7	61 ± 2.9	n = 9	92 ± 1.6	n = 4	62 ± 4.8
Ler	n = 7	74 ± 2	n = 7	45 ± 2.3				
*fil*-5	n = 8	102 ± 3.1	n = 3	52 ± 1.7				
Summary statistics								
	Col-7 vs. *fil-9*				ANOVA, F1,55 = 262.6, P < 0.001			
	Col-7: Early vs. Late meristems				ANOVA, F1,31 = 81, P < 0.001			
	Col-7: temperature (22°C vs. 28°C)				ANOVA, F1,31 = 1.75, P = 0.195			
	*fil-9*: Early vs. Late meristems				ANOVA, F1,28 = 42, P < 0.001			
	*fil-9*: temperature (22°C vs. 28°C)				ANOVA, F1,28 = 6.958, P = 0.0135			
	Ler vs. *fil-5*				ANOVA, F1,21 = 34, P < 0.001			
	Ler: Early vs. Late meristems				ANOVA, F1,12 = 89, P < 0.001			

IM size was significantly larger in *fil-9 *plants (Figures 3B, 3C, 3G, 3H, 3J and 3K), as compared to the Col-7 wild type (Figures 3A, 3F, 3I, Table 3). Interestingly, in both *fil-9 *and wild type plants, early meristems were significantly larger than were late meristems (Table 3). A similar pattern also appeared in another *fil *allele, i.e. *fil-5*, in the L*er *ecotype background (Table 3). *fil-9 *IMs were deformed, with the early meristems being flatter than in the wild type (Figures 3G, 3H compared to 3F). Late *fil-9 *meristems were variable in shape, being either flatter or more swollen than in the wild type (Figures 3J, 3K compared to 3I).

As was shown for the floral phenotype, the sensitivity of *fil-*9 plants to higher temperature was also reflected in an increase of IM size at a growth temperature of 28°C, as compared to growth at 22°C (Table 3). Wild type IMs displayed no sensitivity to high temperature (Table 3).

To test whether *fil-9 *FMs also differed from those of wild type plants, the size of the central zone, defined as the length between opposite sepals, was measured in flowers from both early and late inflorescences. In both cases, *fil-9 *FMs were significantly larger, as compared to those of wild-type plants (Table 4).

**Table 4:** Floral meristem size

	**Early meristem mean ± SE (μm)**		**Late meristem mean ± SE (μm)**	
	
Col-7	n = 5	45 ± 0	n = 3	42 ± 1.7
*fil-9*	n = 4	80 ± 4.1	n = 7	64 ± 2.5

Col-7 vs. *fil-9*: Two sample T-test, t = 6.906255, DF = 17, P < 0.001

The increased size of *fil-9 *meristems could result from either an increase in cell number or cell size. To distinguish between these two possibilities, the number of cells in an area of 966 μm^2 ^was counted (an area corresponding to a circle engulfing approximately the whole wild type meristem; Figures 3L-3N). To facilitate the measurements, the outline of the cells in the meristem was marked by staining the plasma membranes with the lipophilic dye, FM-64, (red) [35] and by L1 layer expression of GFP fused to the plasma-membrane-localized PIN1 protein (green) [36]. The average number of cells in the defined meristematic area did not differ between the *fil-9 *and wild type plants (Figures 3L and 3M), although the *fil-*9 meristem is larger than the wild type. This indicates that the aberrancy of *fil-9 *meristems is not caused by larger cells but rather results from accumulation of more cells in the meristem, as compared to the wild type.

The enlarged *fil-9 *IM and FM are functionally disrupted. This is reflected in the irregular temporal order of emergence of floral primordia on the flanks of the IM, with several floral primordia arising simultaneously, instead of sequentially (Figures 3H (*fil-9*) and 3F (wild type), respectively). In addition, many floral organs emerge concurrently, both within the same and in different whorls within the flower (Figures 3B and 3C).

An additional feature of floral emergence from *fil-9 *IM was the distorted orientation of the flowers towards the axis of the IM, as deduced by the position of the sepals (Figures 3B, 3C and 3E (*fil-9*) compared to 3A and 3D (wild type)). This was accompanied by changes in the order of sepal development. In wild type flowers, the abaxial sepal developed first, followed by the adaxial sepal and finally, the two lateral sepals (Figures 3A and 3D; [37]). However, in *fil-9 *flowers, two sepals, or other organs occupying that position, developed in abaxial lateral positions, with the two other sepals developing later, in the adaxial position (Figures 3B, 3C and 3E).

### The filamentous structures produced by *fil-9 *inflorescence meristem do not replace true flowers

**Figure 4:**
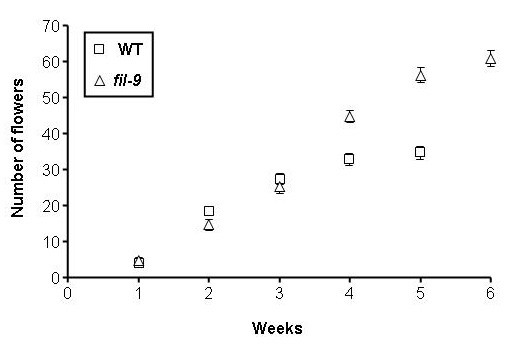
**Number of flowers produced on *fil-9 *and wild type main inflorescences**. The total number of flowers on each inflorescence was counted each week during 6 weeks from bolting. *fil-9*, n = 17; wild type, n = 20. Averages ± SE are graphed.

As previously noted, *fil-9 *defective inflorescence meristems produce several organs simultaneously, with many of these being filamentous organs with no clear identity. Earlier studies on other *fil *alleles reported that the development of such filaments is confined to a defined region along the inflorescence stem [25,26]. Under our growth conditions, we could not find filaments concentrated and/or limited to a specific zone, neither in *fil-9 *nor in two additional alleles (i.e. *fil-5 *and *fil-8*). To assess whether the filaments produced by the *fil-9 *IM have a floral identity, the number of flowers produced by the primary IMs during the course of reproductive development was compared between *fil-9 *and wild type plants. In these measurements, only organs with floral structures but not filaments were counted as flowers. During the first three weeks of flower production, *fil-9 *and wild type IMs produced the same number of flowers, at the same rate (Figure 4). However, in the fourth week, where as the wild type production rate did not change, *fil-9 *floral production accelerated (Figure 4). During the fifth week, wild type floral production sharply declined, while the rate of *fil-9 *floral production was only slightly reduced (Figure 4). Wild type flower production ceased after the fifth week, producing a total of 35 flowers on average, while *fil-9 *IMs proliferated for an additional week, producing a total of 60 flowers on average (Figure 4). This pattern of the proliferative capacity of *fil-9 *IM is similar to that of other reduced fertility mutants [34]. Thus, the production of more flowers by *fil-9 *IMs likely results from the partial self-sterility of *fil-9 *plants and not because of simultaneous emergence of more flowers from the IM. Moreover, the production of the same number of flowers, not including filamentous organs, in *fil-9 *as in wild type during the first three weeks, strongly indicates that the filaments are not considered by the plants as true flowers.

## Discussion

*FIL *encodes a putative transcription factor that is a member of the YABBY family. *YABBY *genes play a demonstrated role in regulating abaxial-adaxial polarity in lateral organs [12,38]. From studies of *YABBY *genes, it is emerging of late that *FIL *also serves a role in shoot meristem organization [24,29]. In the inflorescence, unlike the vegetative parts of the plant, *FIL *functions, at least partially, in non-redundant manner, such that mutations in the gene lead to visually perceptible phenotypes. These phenotypes have been reported for several *fil *alleles in the L*er *background [12,25-27]. In our study, we have characterized a new *fil *allele in the Col background. Through meticulous analysis of reproductive development, we demonstrate a linkage between distortions in *fil *IM and FM and the inflorescence and floral abnormal phenotypes. We further propose an explanation as to how these are causally related, thus highlighting the potential of using *fil *mutants to study mersitem organization and its effect on flower morphogenesis

We have shown that the shape and size of both the IM and FM is altered in *fil-9 *mutant plants (Figure 3), and that this results from an increase in cell number rather than in cell size. These alterations are associated with defects in primordia initiation, such that several floral bud primordia and multiple floral organs can emerge simultaneously. The changes in the FM presumably prevent the establishment of defined whorls and lead to disruption of the orderly allocation of cells to the emerging organs. We propose that as a result of the blurring of the boundaries between different whorls, the *fil-9 *flowers developed the observed defects in organ identity. The hypothesis that *FIL *does not function as a regulator of the expression of organ identity genes per se is supported by the fact that we never observed full organ conversion, while alterations in organ identity occurred only occasionally.

The organ growth defects observed in *fil *mutants are unlikely to be the result of a simple change in meristem size. In mutants affecting meristem size, such as *clavata *or *wuschel*, changes in meristem size indeed lead to an alteration in organ number. The shape and identity of the organs remain, however, unaltered [39-41]. Moreover, although *fil *meristems are larger than are those in wild type plants, the floral defects are mostly displayed in reduced number and size of the floral organs. The variable defects in floral organ formation and organization in *fil-9 *mutants (Figure 2) resemble those produced by mutations in *tousled*, a gene that encodes a protein kinase [42,43]. *tousled clavata *double mutants do not show a restoration of organ patterning, indicating that simply increasing meristem size can not compensate for loss of *tousled *function. This raised the possibility that *TOUSLED *may promote specific cell divisions within the meristem, rather than having a general proliferative effect [43]. Likewise, the high variability in the number and morphology of *fil *floral organs (Figure 2) suggests that disruption of the cell proliferation process occurs within localized areas of activity in the meristem.

The defects we observed are consistent with the suggestion that *FIL *plays a role in regulating the partitioning of the IM and FM into distinct domains. A number of genes have been implicated in establishing meristematic domains by regulating the specification of the CZ, the boundary between proliferating and differentiating cells, or the development of organ primordia at the periphery of the meristem (reviewed in [2]). In addition, auxin flux across the meristem is critical for normal patterning, with feedback between genes regulating auxin distribution and those regulating meristematic domains taking place. This suggests an intimate connection between these pathways [2,44]. For instance, mutations in boundary genes, such as *BOP1 *and *BOP2*, as well as mutations in auxin influx carriers produce inflorescence and floral defects similar to those we observe in *fil-9 *mutants [45-48]. Based on these similarities in phenotype, in conjunction with the defects observed in IM and FM organization, we suggest that *FIL *is required for the appropriate delineation of CZ and PZ boundaries within these meristems. The disorderly emergence of organ primordia in *fil-9 *plants can be explained by disruption of these boundary domains. Many of these organs are, however, filamentous. Note that when these organs were not included when counting the number of flowers produced by *fil-9 *inflorescences, the number of flowers in the mutant was the same as in wild type plants, throughout most of the reproductive phase (Figure 4). Sawa et al. have previously shown that similar filamentous structures in the *fil*-1 mutant express the floral meristem identity gene, *AP1*, but gradually lose the expression of the floral meristem identity gene, *LEAFY *[26]. Together, these results suggest that the filamentous organs produced by *fil *IM have a floral meristem identity, although this is insufficient to maintain floral identity. The disruption of meristem organization can also explain the occasional development of a cryptic bract - a normally arrested organ - in *fil *mutants (Figure 2 and [26]).

Verifying the involvement of *FIL *in the establishment and maintenance of IM boundaries awaits analysis of expression patterns of meristem, primordia and boundary markers, such as *STM, ANT, CUC2 *and *UFO*, in *fil *inflorescences. For example, it was shown by Goldschmidt and colleagues (2008) that altering *FIL *and *YAB3 *expression leads to changes in the expression of *LATERAL SUPPRESSOR*, a gene which is normally expressed at the boundary of organ primordia [24]. In this regard, it could be informative to compare the expression patterns of meristem boundary markers in *fil-9 *which is in the Col background versus other reported *fil *alleles that are in the L*er *background. Differences have been noted in the shape of wild type inflorescence meristems in different *Arabidopsis *ecotypes in terms of height and width though not in the internal organization of layers and zones [49]. It therefore, would be of interest to test whether these different genetic backgrounds differentially sensitize meristem-primordia signaling pathways.

The variable homeotic transformations of floral organs suggest that, similar to IM, *fil *floral whorls boundaries are also likely to be disrupted. It was previously suggested that *fil *might be a direct regulator of floral organ identity genes [25,26]. However, the range of mosaic organs that develop within each flower point towards shifts in the expression domains of the identity genes as a result of misallocation of cells to the different whorls. This could be tested by examining the expression patterns of the floral organ identity genes and whorl boundary genes, such as *SUP*, in *fil *flowers. Previous studies on *fil *alleles have presented analyses of expression of the floral organ identity genes, *AP1*, *APETALA3*, *PISTILLATA *and *AGAMOUS *in *fil *flowers [25,26]. Nevertheless, such analyses were limited to specific types of *fil *abnormal flowers and did not address the wide range of floral organ phenotypes that exist in *fil *mutants. Thus, the complex alteration in expression patterns of the organ identity genes that occurs could not be fully revealed.

In an attempt to explain the formation of mosaic organs in flowers of several *ap2 *and *ag *alleles, Bowman et al. suggest that the primordia of these organs encompass more than a single geographic whorl, resulting in organs composed of cell arising from different whorls [50]. We suggest that in *fil*, whorl boundaries are erratic, resulting in organs with abnormal shapes and mixed identities.

An additional support for the causal relationship between the disrupted meristem organization and the various phenotypes of *fil *floral organs is that these phenotypes are more severe in later-arising inflorescences and flowers (Tables 1, 2). Such acropetal changes in *fil-9 *mutant phenotypes are correlated with changes in meristem size and shape. We have documented that meristems at a late developmental stage are smaller than those at an earlier stage, both in wild type and in *fil *inflorescences (Figure 3, Table 3). It is hence possible that the age-dependent depletion of the meristematic pool intensifies the distortion of the inflorescence and floral mersitems in *fil-9 *plants and thus, their floral phenotypes.

## Conclusions

Through the characterization of a new *fil *allele, *fil-9*, we have re-evaluated the role of *FIL *in the organization of IMs and FMs and in the emergence of reproductive organ primordia. *fil-9 *floral organs are highly variable in terms of organ number, size and morphology. In addition, *fil-9 *plants show disruption of floral whorl boundaries, with floral organs occasionally displaying mixed identities. The high variability of the floral aberrations indicated that they are the result of underlying defects in the meristem. Indeed, both the inflorescence and floral meristems of *fil-9 *plants are larger (i.e. containing more cells) and are distorted, as compared to these meristems in wild type plants, and produce several primordia in a simultaneous rather than a sequential manner. Moreover, the age-dependent decrease in meristem size was correlated with enhanced severity of floral phenotypes. Overall, our results support the role of *FIL *in the organization of the meristem and provide new insight into the relationship between meristem organization and floral form.

## Methods

### Plant materials and growth conditions

The *fil-9 *line was isolated from activation-tagged lines [31] in a Col-7 background. These seeds, as well as wild type Col-7 seeds, were obtained from The Nottingham *Arabidopsis *Stock Center (University of Nottingham, UK). *fil-*5 and *fil-*8 seeds were a kind gift from Yuval Eshed, Weizmann Institute of Science, Rehovot, Israel. *pPIN1::PIN1-GFP *seeds were plated on a mixture of soil (sphagnum peat and tuff) and perlite (2:1) and grown under either a 16 h light/8 h dark cycle or constant light (pPIN1::PIN1-GFP) at 22°C or 28°C, as indicated.

For the characterization of the *fil-9 *phenotype, a total of 70 early-arising (flowers 1-10) and 162 late-arising (flower 11 and later) flowers from 7 plants grown at 22°C were analyzed. A total of 172 flowers from 3 *fil-9 *plants grown at 28°C were analyzed for the temperature-dependency comparison. To measure the number of flowers produced on *fil-9 *plants, as compared to the wild type, the main inflorescences of 17 *fil-9 *and 20 wild type plants were analyzed during 6 weeks from bolting.

### Light, scanning and confocal electron microscopy

Fresh tissue was dissected and examined with a SMZ800 dissecting microscope (Nikon) and pictures were taken with an attached Coolpix 4500 digital camera (Nikon).

For scanning electron microscope (SEM), flowers were fixed in 3.7% formaldehyde, 50% ethanol, and 5% acetic acid for 8 h and dehydrated in a graded ethanol series. Dehydrated flowers were critical point dried in liquid CO_2 _and sputter-coated with gold palladium. Specimens were analyzed and photographed with a JSM5610LV scanning electron microscope (Jeol, Japan).

Fresh meristems were dissected into GM medium and incubated with FM4-64 (Invitrogen™) at a final concentration of 10 μM for 10-20 min. All imaging was done using a Zeiss LSM 510 Meta confocal microscopy system using a waterdipping 40× objective. GFP was excited with a 488 nm Argon laser, with emission detection through the meta-channel at 497 to 550 nm.

### Measurements of meristem size and cell number

Meristem size measurements were performed from the SEM scans. The FM measurements were performed in developing flowers, containing only sepal primordia, with mesritem size being determined as the length between opposite sepals.

Cell number was counted in a circle of 966 μm^2^, an area that covered most of the wild type inflorescence meristem. We used the pPIN1::PIN1-GFP construct [36] (in the background of the wild type or *fil-9 *plants) as a marker for the meristematic L1 cell layer.

For the statistical analysis, we employed two-way Analysis of Variance (ANOVA) or two sample T-test, as indicated. Analyses were done using SYSTAT v. 11 (SYSTAT Software, San Jose, CA).

### DNA and RNA extraction and RT-PCR

Plant DNA was extracted using the CTAB extraction method [51]. Mapping of the T-DNA insertion in *fil-9 *plants was performed by PCR amplification using a T-DNA specific primer (tsp1 5'-ACG ACG GAT CGT AAT TTG TCG T-3') and a *FIL *specific primer (infilrv 5'-TCT GTG GCT TAT ATC AGG ATT ACC AG-3').

Total RNA was extracted using an EZ total RNA isolation kit (Biological Industries, Beit Haemek, Israel). cDNA was synthesized using a Reverse-iT 1^st ^Strand Synthesis Kit (ABgene, Epsom, UK). Semi-quantitative RT-PCR reactions were performed using *FIL *specific primers (forward primers FILP1 5'-TCT CCT TCC GAC CAT CTC TG-3' or FILP3 5'-CTC CAG AGA AAA GAC AGA GAG TC-3', and reverse primers FILP2 5'-AGA TTG GTA CAG CAA CCA CAT C-3' or FILP4 5'-CCA ACG TTA GCA GCT GCA GGA-3'), and *Tubulin4 *(*At5g44340*) primers (TUB4F 5'-CCG GTC AAT ACG TCG GCG-3' and TUB4R 5'-TCA GAG ACC TTA GGA GAA GG-3') as control.

## Authors' contributions

NL conducted the genetic molecular analyses to map the mutation and all the scanning electron and confocal microscopy studies. NN participated in the isolation of the mutant, designing the study, interpreting the data and writing the manuscript, RB did the mutant floral phenotyping, MZ designed and supervised the study and wrote the manuscript. All authors read and approved the final manuscript.

## References

[B1] Baurle I Laux T Apical meristems: the plant's fountain of youth BioEssays 2003 25 10 961 970 10.1002/bies.10341 14505363

[B2] Rast MI Simon R The meristem-to-organ boundary: more than an extremity of anything Curr Opin Genet Dev 2008 18 4 287 294 10.1016/j.gde.2008.05.005 18590819

[B3] Carraro N Peaucelle A Laufs P Traas J Cell differentiation and organ initiation at the shoot apical meristem Plant Mol Biol 2006 60 6 811 826 10.1007/s11103-005-2761-6 16724254

[B4] Blazquez MA Ferrandiz C Madueno F Parcy F How floral meristems are built Plant Mol Biol 2006 60 6 855 870 10.1007/s11103-006-0013-z 16724257

[B5] Schoof H Lenhard M Haecker A Mayer KFX Jurgens G Laux T The stem cell population of *Arabidopsis *shoot meristems is maintained by a regulatory loop between the *CLAVATA *and *WUSCHEL *genes Cell 2000 100 635 644 10.1016/S0092-8674(00)80700-X 10761929

[B6] Lenhard M Laux T Stem cell homeostasis in the *Arabidopsis *shoot meristem is regulated by intercellular movement of CLAVATA3 and its sequestration by CLAVATA1 Development 2003 130 14 3163 3173 10.1242/dev.00525 12783788

[B7] Scofield S Murray JA *KNOX *gene function in plant stem cell niches Plant Mol Biol 2006 60 6 929 946 10.1007/s11103-005-4478-y 16724262

[B8] Krizek BA Ectopic expression of *AINTEGUMENTA *in *Arabidopsis *plants results in increased growth of floral organs Dev Genet 1999 25 3 224 236 10.1002/(SICI)1520-6408(1999)25:3<224::AID-DVG5>3.0.CO;2-Y 10528263

[B9] Mizukami Y Fischer RL Plant organ size control *AINTEGUMENTA *regulates growth and cell numbers during organogenesis Proc Natl Acad Sci USA 2000 97 2 942 947 10.1073/pnas.97.2.942 10639184 PMC15435

[B10] Eshed Y Baum SF Perea JV Bowman JL Establishment of polarity in lateral organs of plants Curr Biol 2001 11 16 1251 1260 10.1016/S0960-9822(01)00392-X 11525739

[B11] Waites R Selvadurai HR Oliver IR Hudson A The *PHANTASTICA *gene encodes a MYB transcription factor involved in growth and dorsoventrality of lateral organs in *Antirrhinum* Cell 1998 93 5 779 789 10.1016/S0092-8674(00)81439-7 9630222

[B12] Siegfried KR Eshed Y Baum SF Otsuga D Drews GN Bowman JL Members of the *YABBY *gene family specify abaxial cell fate in *Arabidopsis* Development 1999 126 18 4117 4128 10457020 10.1242/dev.126.18.4117

[B13] Waites R Hudson A The *HANDLEBARS *gene is required with *PHANTASTICA *for dorsoventral asymmetry of organs and for stem cell activity in *Antirrhinum* Development 2001 128 11 1923 1931 11493516 10.1242/dev.128.11.1923

[B14] Stuurman J Jaggi F Kuhlemeier C Shoot meristem maintenance is controlled by a *GRAS*-gene mediated signal from differentiating cells Genes Dev 2002 16 17 2213 2218 10.1101/gad.230702 12208843 PMC186665

[B15] Krizek BA Fletcher JC Molecular mechanisms of flower development an armchair guide Nat Rev Genet 2005 6 9 688 698 10.1038/nrg1675 16151374

[B16] Sakai H Medrano LJ Meyerowitz EM Role of *SUPERMAN *in maintaining *Arabidopsis *floral whorl boundaries Nature 1995 378 199 201 10.1038/378199a0 7477325

[B17] Levin JZ Meyerowitz EM *UFO*: an *Arabidopsis *gene involved in both floral meristem and floral organ development Plant Cell 1995 7 529 548 10.1105/tpc.7.5.529 7780306 PMC160802

[B18] Wilkinson MD Haughn GW *UNUSUAL FLORAL ORGANS *controls meristem identity and organ primordia fate in *Arabidopsis* Plant Cell 1995 7 1485 1499 10.1105/tpc.7.9.1485 12242408 PMC160975

[B19] Aida M Ishida T Fukaki H Fujisawa H Tasaka M Genes involved in organ separation in *Arabidopsis*: an analysis of the cup-shaped cotyledon mutant Plant Cell 1997 9 6 841 857 10.1105/tpc.9.6.841 9212461 PMC156962

[B20] Takada S Hibara K Ishida T Tasaka M The *CUP-SHAPED COTYLEDON1 *gene of *Arabidopsis *regulates shoot apical meristem formation Development 2001 128 7 1127 1135 11245578 10.1242/dev.128.7.1127

[B21] Baker CC Sieber P Wellmer F Meyerowitz EM The early extra petals1 mutant uncovers a role for microRNA miR164c in regulating petal number in *Arabidopsis* Curr Biol 2005 15 4 303 315 10.1016/j.cub.2005.02.017 15723790

[B22] Watanabe K Okada K Two discrete cis elements control the Abaxial side-specific expression of the *FILAMENTOUS FLOWER *gene in *Arabidopsis* Plant Cell 2003 15 11 2592 2602 10.1105/tpc.015214 14555697 PMC280563

[B23] Gordon SP Heisler MG Reddy GV Ohno C Das P Meyerowitz EM Pattern formation during de novo assembly of the *Arabidopsis *shoot meristem Development 2007 134 19 3539 3548 10.1242/dev.010298 17827180

[B24] Goldshmidt A Alvarez JP Bowman JL Eshed Y Signals derived from *YABBY *gene activities in organ primordia regulate growth and partitioning of *Arabidopsis *shoot apical meristems Plant Cell 2008 20 5 1217 1230 10.1105/tpc.107.057877 18469164 PMC2438466

[B25] Chen Q Atkinson A Otsuga D Christensen T Reynolds L Drews GN The *Arabidopsis **FILAMENTOUS FLOWER *gene is required for flower formation Development 1999 126 12 2715 2726 10331982 10.1242/dev.126.12.2715

[B26] Sawa S Ito T Shimura Y Okada K *FILAMENTOUS FLOWER *controls the formation and development of *Arabidopsis *inflorescences and floral meristems Plant Cell 1999 11 1 69 86 10.1105/tpc.11.1.69 9878633 PMC144087

[B27] Kumaran MK Ye D Yang W-C Griffith ME Chaudhury AM Sundaresan V Molecular cloning of *ABNORMAL FLORAL ORGANS*: a gene required for flower development in *Arabidopsis* Sex Plant Reprod 1999 12 118 122 10.1007/s004970050180

[B28] Chen C Xu Y Zeng M Huang H Genetic control by *Arabidopsis *genes *LEUNIG *and *FILAMENTOUS FLOWER *in gyneocium fusion Journal of Plant Research 2001 114 465 469 10.1007/PL00014012

[B29] Stahle MI Kuehlich J Staron L von Arnim AG Golz JF YABBYs and the Transcriptional Corepressors LEUNIG and LEUNIG_HOMOLOG Maintain Leaf Polarity and Meristem Activity in *Arabidopsis* Plant Cell 2009 19837869 10.1105/tpc.109.070458 PMC2782291

[B30] Garcia D Collier SA Byrne ME Martienssen RA Specification of leaf polarity in *Arabidopsis *via the trans-acting siRNA pathway Curr Biol 2006 16 9 933 938 10.1016/j.cub.2006.03.064 16682355

[B31] Weigel D Ahn JH Blazquez MA Borevitz JO Christensen SK Fankhauser C Ferrandiz C Kardailsky I Malancharuvil EJ Neff MM Activation tagging in *Arabidopsis* Plant Physiol 2000 122 4 1003 1013 10.1104/pp.122.4.1003 10759496 PMC1539247

[B32] Kumaran MK Bowman JL Sundaresan V *YABBY *polarity genes mediate the repression of *KNOX *homeobox genes in *Arabidopsis* Plant Cell 2002 14 11 2761 2770 10.1105/tpc.004911 12417699 PMC152725

[B33] Gasteiger E Gattiker A Hoogland C Ivanyi I Appel RD Bairoch A ExPASy: The proteomics server for in-depth protein knowledge and analysis Nucleic Acids Res 2003 31 13 3784 3788 10.1093/nar/gkg563 12824418 PMC168970

[B34] Hensel LL Nelson MA Richmond TA Bleecker AB The fate of inflorescence meristems is controlled by developing fruits in *Arabidopsis* Plant Physiol 1994 106 3 863 876 10.1104/pp.106.3.863 7824655 PMC159609

[B35] Reddy GV Live-imaging stem-cell homeostasis in the *Arabidopsis *shoot apex Curr Opin Plant Biol 2008 11 1 88 93 10.1016/j.pbi.2007.10.012 18069047

[B36] Wisniewska J Xu J Seifertova D Brewer PB Ruzicka K Blilou I Rouquie D Benkova E Scheres B Friml J Polar PIN Localization Directs Auxin Flow in Plants Science 2006 312 5775 883 10.1126/science.1121356 16601151

[B37] Smyth DR Bowman JL Meyerowitz EM Early flower development in *Arabidopsis* Plant Cell 1990 2 755 767 10.1105/tpc.2.8.755 2152125 PMC159928

[B38] Eshed Y Izhaki A Baum SF Floyd SK Bowman JL Asymmetric leaf development and blade expansion in *Arabidopsis *are mediated by *KANADI *and *YABBY *activities Development 2004 131 12 2997 3006 10.1242/dev.01186 15169760

[B39] Clark SE Running MP Meyerowitz EM *CLAVATA3 *is a specific regulator of shoot and floral meristem development affecting the same processes as *CLAVATA1* Development 1995 121 2057 2067

[B40] Clark SE Running MP Meyerowitz EM *CLAVATA1 *a regulator of meristem and flower development in *Arabidopsis* Development 1993 119 2 397 418 8287795 10.1242/dev.119.2.397

[B41] Laux T Mayer KFX Berger J Jurgen G The *WUSCHEL *gene is required for shoot and floral meristem integrity in *Arabidopsis* Development 1996 122 87 96 8565856 10.1242/dev.122.1.87

[B42] Roe JL Rivin CJ Sessions RA Feldmann KA Zambryski PC The *TOUSLED *gene in A. thaliana encodes a protein kinase homolog that is required for leaf and flower development Cell 1993 75 5 939 950 10.1016/0092-8674(93)90537-Z 8252629

[B43] Roe JL Nemhauser JL Zambryski PC *TOUSLED *participates in apical tissue formation during gynoecium development in *Arabidopsis* Plant Cell 1997 9 3 335 353 10.1105/tpc.9.3.335 9090879 PMC156922

[B44] Bowman JL Floyd SK Patterning and polarity in seed plant shoots Annu Rev Plant Biol 2008 59 67 88 10.1146/annurev.arplant.57.032905.105356 18031217

[B45] Hepworth SR Zhang Y McKim S Li X Haughn GW *BLADE-ON-PETIOLE*-dependent signaling controls leaf and floral patterning in *Arabidopsis* Plant Cell 2005 17 5 1434 1448 10.1105/tpc.104.030536 15805484 PMC1091766

[B46] Norberg M Holmlund M Nilsson O The *BLADE ON PETIOLE *genes act redundantly to control the growth and development of lateral organs Development 2005 132 9 2203 2213 10.1242/dev.01815 15800002

[B47] Ha CM Jun JH Nam HG Fletcher JC *BLADE-ON-PETIOLE *1 and 2 control *Arabidopsis *lateral organ fate through regulation of LOB domain and adaxial-abaxial polarity genes Plant Cell 2007 19 6 1809 1825 10.1105/tpc.107.051938 17601823 PMC1955725

[B48] Bainbridge K Guyomarc'h S Bayer E Swarup R Bennett M Mandel T Kuhlemeier C Auxin influx carriers stabilize phyllotactic patterning Genes Dev 2008 22 6 810 823 10.1101/gad.462608 18347099 PMC2275433

[B49] Laufs P Grandjean O Jonak C Kieu K Traas J Cellular parameters of the shoot apical meristem in *Arabidopsis* Plant Cell 1998 10 8 1375 1390 10.1105/tpc.10.8.1375 9707536 PMC144064

[B50] Bowman JL Smyth DR Meyerowitz EM Genetic interactions among floral homeotic genes of *Arabidopsis* Development 1991 112 1 20 1685111 10.1242/dev.112.1.1

[B51] McDonald BA Martinez JP Restriction fragment length polymorphisms in Septoria tritici occur at a high frequency Current Genetics 1990 17 2 133 138 10.1007/BF00312858

[B52] Bowman JL The *YABBY *gene family and abaxial cell fate Curr Opin Plant Biol 2000 3 1 17 22 10.1016/S1369-5266(99)00035-7 10679447

